# Prevalence of obesity and associated sociodemographic and lifestyle factors in Ecuadorian children and adolescents

**DOI:** 10.1038/s41390-024-03342-w

**Published:** 2024-06-24

**Authors:** José Francisco López-Gil, Sitong Chen, Rubén López-Bueno, Hector Gutiérrez-Espinoza, Miguel Angelo Duarte Junior, Pablo Galan-Lopez, José Luis Palma-Gamiz, Lee Smith

**Affiliations:** 1 One Health Research Group, Universidad de Las Américas, Quito, Ecuador; 2 Department of Communication and Education, Universidad Loyola Andalucía, Seville, Spain; 3 Institute for Health and Sport, Victoria University, Melbourne, VIC Australia; 4 Department of Physical Medicine and Nursing, University of Zaragoza, Zaragoza, Spain; 5 Department of Preventive Medicine and Public Health, School of Medicine, Universidad Autónoma de Madrid, Madrid, Spain; 6 Spanish Institute of Lifestyle Medicine, Madrid, Spain; 7 Centre for Health Performance and Wellbeing, Anglia Ruskin University, Cambridge, UK

## Abstract

**Background:**

Given the increasing prevalence of obesity in young people in Ecuador, there is a need to understand the factors associated with this condition. The aim of this study was to assess the prevalence of obesity in Ecuadorian children and adolescents aged 5–17 years and identify its associated sociodemographic and lifestyle factors.

**Methods:**

This cross-sectional study was conducted using data from the *Encuesta Nacional de Salud y Nutrición* (ENSANUT-2018). The final sample consisted of 11,980 participants who provided full information on the variables of interest.

**Results:**

The prevalence of obesity was 12.7%. A lower odd of having obesity was observed for adolescents; for those with a breadwinner with an educational level in middle/high school or higher; for each additional day with 60 or more minutes of daily moderate-to-vigorous physical activity; and for those with greater daily vegetable consumption (one, two, or three or more servings). Conversely, there were greater odds of obesity in participants from families with medium, poor, and very poor wealth and those from the coast and insular region.

**Conclusions:**

The high prevalence of obesity in Ecuadorian children and adolescents is a public health concern. Sociodemographic and lifestyle behavior differences in young people with obesity should be considered when developing specific interventions.

**Impact:**

- As the prevalence of obesity among children and adolescents increases in Latin America, with a particular focus on Ecuador, it becomes crucial to delve into the factors linked to this condition and identify the most successful strategies for its mitigation.
- The elevated prevalence of obesity among young individuals in Ecuador raises significant public health concerns.
- To develop targeted interventions, it is crucial to account for sociodemographic variables and lifestyle behaviors that contribute to obesity in this population.

## Introduction

Pandemic obesity in children and adolescents is a public health problem in high-income countries and is increasingly prevalent in low and middle-income countries (LMICs).^1,2^ The World Obesity Federation predicts that by 2025, there will be approximately 206 million children and adolescents with obesity, and this number will increase to approximately 254 million by 2030.^3^ This public health problem has aroused policymakers’ concerns across the world.^1^ For LMICs, data from Hajri et al.^4^ suggested a pooled prevalence of obesity was 8.1% (95% confidence interval [CI] 6.9 to 9.3), 10.7% (95% CI 9.6 to 11.7) and 10.5% (95% CI 9.2 to 11.8) for Ecuadorian preschoolers, children and adolescents, respectively. Similarly, the most recent study, the *Encuesta Nacional de Salud y Nutrición* (ENSANUT)-2018, in Ecuador revealed that the prevalence of obesity was 14.8% (based on the World Health Organization classification).^5^ According to the same classification, 7.0% of the adolescents (aged 12 to 19 years) had obesity.

Obesity develops when a series of genetic, epigenetic, behavioral, environmental and sociodemographic factors impact body weight through two pathways: energy homeostasis and cognitive-emotional control.^6^ Regarding sociodemographic factors, certain age,^1^ sex,^1^ socioeconomic status,^7,8^ or area of residence (i.e., urban/rural)^9^ inequalities have been found for young people with obesity. Moreover, studies have indicated that disparities in obesity may also be influenced by factors such as education level and race/ethnicity,^10^ highlighting the multifaceted nature of sociodemographic factors in explaining obesity.^11^ Despite these inequalities, there is a gap across the studies conducted in high-income countries versus those in LMICs.^12^

On the other hand, several studies have indicated that promoting and sustaining healthy lifestyle behaviors could serve as an effective approach against obesity among young people.^13–15^ For instance, meeting the 24-h movement recommendations (i.e., physical activity, recreational screen time, and sleep duration) has been cross-sectionally associated with lower overall obesity-related indicators in children and adolescents.^13^ Regarding diet, a systematic review by Liberali et al.^15^ reported that a healthy diet could be effective in reducing the risk of developing childhood obesity. Similarly, children and adolescents with an insufficient consumption of fruits and vegetables and a high consumption of unhealthy foods such as snacks and sweets, in addition to other unhealthy lifestyle factors, had higher probabilities of having overweight or obesity.^16^ This is concerning because the consumption of calorie-dense, high-fat, and low-fiber foods during adolescence is associated with overweight and obesity later in life.^17^ Finally, another lifestyle-related factor that has been associated with obesity is oral health, with children with excess weight (i.e., overweight or obesity) showing higher probabilities of having dental caries than their counterparts with normal weight.^18^

Given the increasing prevalence of obesity in children and adolescents in Latin America^1^ (especially in Ecuador^4^), there is a need to understand the factors associated with obesity and the most effective ways to address it.^19^ Accumulating knowledge based on contextually relevant data is key for developing effective public health policies.^20^ Therefore, the aim of this study was twofold: first, to assess the prevalence of obesity; second, to identify the sociodemographic and lifestyle factors related to obesity in Ecuadorian children and adolescents aged 5 to 17 years.

## Materials and methods

### Study design and population sample

A cross-sectional study was conducted using data from the ENSANUT-2018.^21^ The *Instituto Nacional de Estadística y Censos* (INEC) conducted this survey with the primary objective of generating indicators related to the health status and nutrition of the Ecuadorian population. The purpose was to assess and formulate public policies. ENSANUT-2018, a nationally representative survey, aimed to update the information collected in 2012. The survey covered geographical areas at the national, regional, and provincial levels. Trained interviewers, organized into teams, visited selected households during the data collection process. Before conducting the survey, all participants gave informed consent and agreed to participate. Since this was a secondary analysis with deidentified data, approval from an Institutional Review Board was not needed.

The target population for ENSANUT-2018 included all members of households across the country. Specifically, the study population was divided based on the forms used for data collection, as detailed below: (a) household; (b) women of reproductive age; (c) sexual and reproductive health (for men aged 12 years and older); (d) risk factors (children aged 5 to under 18 years old); and (e) child development (for children under 5 years old nationwide).

To choose the sample, a two-stage and stratified probabilistic sampling approach was employed, encompassing a total of 2591 conglomerates and 46,638 households selected across Ecuador. This sampling method covered both urban and rural areas within the 24 provinces of the country. Of the 46,638 homes initially selected and visited, 43,097 were ultimately included, resulting in a national coverage rate of 92.4%. The final sample for this study included 11,980 participants aged 5 to 17 years with complete information on the variables of interest. Figure S1 shows the flow chart of the participant selection process.

### Procedures

#### Independent variables

##### Sociodemographic variables

The age and sex of the participants were reported by parents or guardians. The educational level of the main breadwinner was classified as “none or literacy center”, “primary education”, “middle/high school”, or “higher education”. For further analyses, we recoded these groups as “primary education or lower” or “middle/high school or higher”. The identification of participants’ race/ethnicity had the following response options: “Indigenous”, “Afro-Ecuadorian”, “Mestizo”, “White”, and “*Montubio* or other”. The area of residence (i.e., urban or rural) was also considered. Urban areas are defined as population centers with a population of 2000 or more inhabitants according to the recommendations of the Andean Community of Nations, regardless of whether they are administrative centers. Population centers with fewer than 2000 inhabitants were considered rural areas. The different Ecuadorian regions were categorized as follows: highlands, coast, amazon, or insular region. Additionally, the estimation of the wealth index in this study utilized variables outlined in a prior research paper that focused on constructing a wealth index using ENSANUT-2018.^22^ The variables pertaining to ownership of goods or services were binary, with 0 indicating that the household did not possess the item and 1 indicating that it did (e.g., refrigerator, computer, washing machine, blender, microwave, iron, TV, DVD, heater, telephone line, car or van, internet access, cable TV access). Regarding household characteristics, access to basic services was represented as ordinal variables with three to four categories each (e.g., water source, type of floor, roof material, toilet facilities, and number of rooms). A weight (factor score) was assigned to each asset through principal component analysis following the method applied by Mera et al.^22^ Each household received a score for each asset, and these scores were summed for each household. Using the results that incorporated sample weights, households were categorized into quintiles based on their total household score as follows: “very poor”, “poor”, “medium”, “rich”, and “very rich”.

##### Lifestyle variables

Lifestyle questions were self-reported by parents or legal guardians in the case of participants younger than 10 years old and by the participants themselves when they were aged 10 to 17 years. Active commuting was assessed by the following question: “During the last week that your child attended school, how many days did he or she walk or ride a bicycle from home to school or from school to home?” (response options: from 0 to 5 days a week). The daily minutes of physical education were computed through the following three questions: (a) “How many days a week does your child attend physical education classes?” (response options: from 0 to 5 days a week); (b) “How many hours a week does your child attend physical education classes?” (response options: numeric); and (c) How many minutes of class time does your child attend each physical education class? (response options: numeric). Moderate-to-vigorous physical activity was measured with the following question: “During the last 7 days, how many days did your child engage in physical activity for at least 60 min a day (excluding physical education classes at school)?” (response options: from 0 to 7 days a week). Subsequently, the prevalence of meeting the World Health Organization physical activity recommendations was computed.^23^ Sedentary behavior was assessed by the following question: “During a normal day, how much time do you spend sitting or lying down while watching television, playing video games on the computer, chatting with friends, or engaging in other activities that require sitting, such as chatting, browsing the internet, or sending emails?”. Participants were asked to indicate the exact number of hours and minutes they engaged in these behaviors.

The frequency of certain eating habits was assessed by different questions. For instance, fruit, vegetable, and soft drink consumption was assessed as follows: “How many times a week does your child eat or drink fruits/vegetables/soft drinks?” (response options: from 0 to 7 days a week). These questions were subsequently asked about the exact number of servings consumed on those days as follows: “And on one of those days, how many fruits/vegetables/soft drinks or servings of fruit/vegetables/soft drinks does your child eat/drink?”. Fast food consumption was assessed using the following question: “During the past seven days, how many days did your child eat at fast food places or chains that sell French fries, hamburgers, tacos, hot dogs, pizza, etc.?” (response options: from 0 to 7 days a week). The consumption of ultra-processed food was evaluated as follows: “During the last 7 days, on how many days has your child eaten processed products such as crackers, cookies, chips, corn snacks, chocolates, etc.?” (response options: from 0 to 7 days a week).

Oral hygiene was assessed as follows: “How often does your child brush his/her teeth? (response options: never, once a week, two or three times a week, once a day, or two or more times a day). For further analyses, we combined these response options into two groups: “not daily” (never, once a week, or two or three times a week) or “daily” (once a day or two or more times a day).

### Dependent variables

Body weight was measured using a portable scale, and height was measured using a portable measuring rod. These anthropometric variables were measured on three occasions during the same visit on the same day. The measurements were taken by personnel trained by the INEC prior to the study. To obtain weight (in kg) and height (in cm), the mean of the three measurements taken for each patient was considered. Body mass index was computed using the following formula: body weight (kg) ⁄ (height (in square meters). Subsequently, body mass index *z* scores were calculated following the World Health Organization criteria^24,25^; subsequently, the prevalence of obesity (+2 standard deviations) was determined.

### Statistical analysis

Statistical analyses were performed using R software (version 4.3.2) and RStudio (2023.09.1 + 494) by Posit in Boston, Massachusetts, USA. Descriptive data are presented as absolute and relative frequencies for categorical variables and means and standard deviations for continuous variables. The “survey” package in R was used to perform design-based analyses that accounted for stratification, clustering, and unequal probability of selection. Therefore, survey-weighted generalized linear models were generated to obtain odds ratios (ORs) with 95% CIs for obesity according to the abovementioned sociodemographic and lifestyle variables. For these analyses, a quasibinomial distribution was applied to avoid warning about the success of noninteger numbers. Univariate (considering the sociodemographic and lifestyle variables individually) and multivariate (including all variables) models were carried out. Additional analyses were performed by dividing the sample by sex and age group. Lastly, statistical significance was established at a *p* value less than 0.05.

## Results

**Fig. 1:**
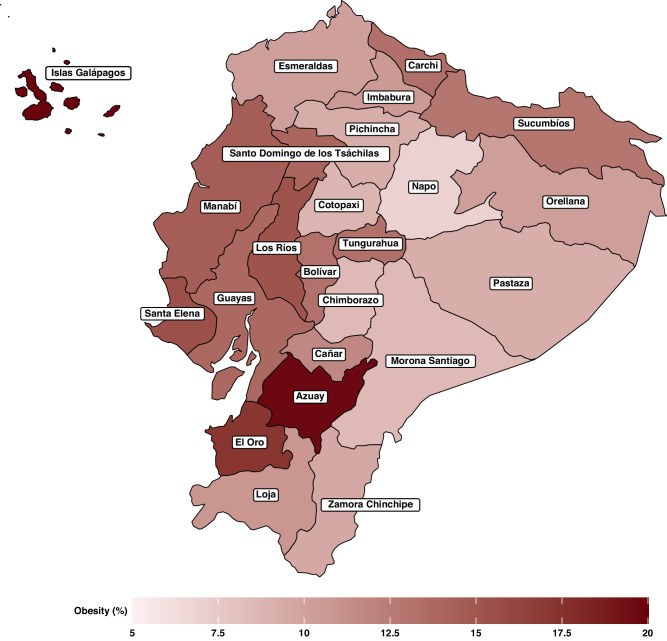
Prevalence of obesity in Ecuadorian children and adolescents (aged 5 to 17 years) according to different regions.

Figure 1 displays the prevalence of obesity in children and adolescents aged 5 to 17 years in the different Ecuadorian regions. The highest prevalence was found in *Islas Galápagos* (19.9%). Conversely, the lowest prevalence was found in *Napo* (6.9%). Table 1 shows the characteristics of the study participants. Overall, the prevalence of obesity was 12.7%.

**Table 1:** Descriptive data of the study participants (*N* = 11,970).

Variables		Total
Sociodemographic		
Sex	Boys	6320 (52.8)
	Girls	5650 (47.2)
Age	Mean ± SD	10.8 (3.6)
Age group	Children (5 to 11 y)	6739 (56.3)
	Adolescents (12 to 17 y)	5231 (43.7)
Educational level of the main breadwinner	Primary education or lower	10,274 (85.8)
	Middle/high school or higher	1696 (14.2)
Wealth family index status	Very rich	1737 (14.5)
	Rich	2293 (19.2)
	Medium	2549 (21.3)
	Poor	2685 (22.4)
	Very poor	2706 (22.6)
Race/ethnicity	Indigenous	1309 (10.9)
	Afro-Ecuadorian	460 (3.8)
	Mestizo	9602 (80.2)
	White	156 (1.3)
	*Montubio* or Other	443 (3.7)
Area of residence	Urban	7797 (65.1)
	Rural	4173 (34.9)
Region	Highlands	4621 (38.6)
	Coast	4453 (37.2)
	Amazon	2474 (20.7)
	Insular region	422 (3.5)
Lifestyle		
Active commuting (days)	Mean ± SD	2.7 ± 2.5
Daily active commuting	No	5728 (47.9)
	Yes	6242 (52.1)
Daily physical education (minutes)	Mean ± SD	34.2 ± 27.3
Daily physical education status	0 h	-
	1 h	2693 (22.5)
	2 h	6244 (52.2)
	3 or more hours	3033 (25.3)
Weekly MVPA with 60 or more minutes (days)	Mean ± SD	2.6 ± 2.3
Meeting PA recommendations^a^	No	10,640 (88.9)
	Yes	1330 (11.1)
Daily sedentary behavior (minutes)	Mean ± SD	118.9 ± 72.3
Daily sedentary behavior	No hour	90 (0.8)
	1 h	4491 (37.5)
	2 h	3839 (32.1)
	3 or more hours	3550 (29.7)
Daily fruit consumption status	No serving	37 (0.3)
	1 serving	974 (8.1)
	2 servings	1965 (16.4)
	3 or more servings	8994 (75.1)
Daily vegetables consumption status	No serving	42 (0.4)
	1 serving	1538 (12.8)
	2 servings	2154 (18.0)
	3 or more servings	8236 (68.8)
Daily soft drinks consumption (servings)	No serving	130 (1.1)
	1 serving	3411 (28.5)
	2 servings	2893 (24.2)
	3 or more servings	5536 (46.2)
Weekly fast-food consumption (days)	Mean ± SD	1.2 ± 1.4
Weekly processed food consumption (days)	Mean ± SD	2.3 ± 1.8
Tooth brushing status	Nondaily	629 (5.3)
	Daily	11,341 (94.7)
Anthropometric		
Obesity^b^	No	10,445 (87.3)
	Yes	1525 (12.7)

*MVPA* moderate-to-vigorous physical activity, *PA* physical activity, *SD* standard deviation.

^a^According to the World Health Organization guidelines (22).

^b^According to the World Health Organization criteria (23,24). *MVPA* moderate-to-vigorous physical activity; *PA* physical activity; *SD* standard deviation.

Survey-weighted generalized linear models with ORs and 95% CIs for obesity are shown in Table 2. According to multivariate analysis, the odds of obesity were lower for adolescents than for children (OR = 0.51, 95% CI 0.41 to 0.64, *p* < 0.001); for girls than for boys (OR = 0.65, 95% CI 0.53 to 0.80, *p* < 0.001); for participants with a breadwinner with an educational level in middle/high school or higher than for those with a primary education or lower (OR = 0.61, 95% CI 0.39 to 0.95, *p* = 0.027); for each additional day, the participants had 60 or more minutes of daily moderate-to-vigorous physical activity (OR = 0.95, 95% CI 0.90 to 0.99, *p* = 0.022); and for participants with a daily vegetable consumption of one serving per day (OR = 0.25, 95% CI = 0.08 to 0.77, *p* = 0.016), two servings (OR = 0.24, 95% CI 0.08 to 0.76, *p* = 0.015) or three or more servings (OR = 0.30, 95% CI = 0.10 to 0.93, *p* = 0.037) than for those with no serving. Conversely, we detected greater odds of having obesity in participants with medium (OR = 1.58, 95% CI 1.10 to 2.27, *p* = 0.014), poor (OR = 1.65, 95% CI 1.13 to 2.41, *p* = 0.009), and very poor wealth family status (OR = 1.99, 95% CI 1.32 to 3.00, *p* = 0.001)  in comparison with participants with very rich wealth family status. Furthermore, participants from the coast (OR = 1.34 95% CI 1.07 to 1.69, *p* = 0.011) and insular region (OR = 2.02, 95% CI 1.42 to 2.88, *p* < 0.001) were more likely to have obesity than participants from the highlands were. Analyses by sex and age group can be found in the Supplementary Material (sex: Tables S1 and S2; age group: Tables S3 and S4). Overall, the results remained constant regardless of sex or age group.

**Table 2:** Survey-weighted generalized linear models to evaluate the probability of having obesity in the Ecuadorian 5- to 17-year-old population of the *Encuesta Nacional de Salud y Nutrición*, 2018.

Predictors		Dependent variable (obesity)	
		Univariate analysis	Multivariate analysis
		OR (95% CI, *p* value)	OR (95% CI, *p* value)
Age group	Children (5 to 11 years)	Reference	Reference
	Adolescents (12 to 17 years)	0.46 (0.38–0.57, *p* < 0.001)	0.51 (0.41–0.64, *p* < 0.001)
Sex	Boys	Reference	Reference
	Girls	0.70 (0.58–0.86, *p* = 0.001)	0.65 (0.53–0.80, *p* < 0.001)
Educational level of the main breadwinner	Primary education or lower	Reference	Reference
	Middle/high school or higher	0.41 (0.27–0.60, *p* < 0.001)	0.61 (0.39–0.95, *p* = 0.027)
Wealth family index status	Very rich	Reference	Reference
	Rich	1.10 (0.79–1.55, *p* = 0.572)	1.13 (0.80–1.61, *p* = 0.490)
	Medium	1.45 (1.04–2.04, *p* = 0.030)	1.58 (1.10–2.27, *p* = 0.014)
	Poor	1.51 (1.08–2.12, *p* = 0.016)	1.65 (1.13–2.41, *p* = 0.009)
	Very poor	1.69 (1.20–2.39, *p* = 0.003)	1.99 (1.32–3.00, *p* = 0.001)
Race/ethnicity	Indigenous	Reference	Reference
	Afro-Ecuadorian	1.02 (0.59–1.75, *p* = 0.953)	0.87 (0.48–1.57, *p* = 0.636)
	Mestizo	1.43 (1.02–2.01, *p* = 0.039)	1.13 (0.76–1.66, *p* = 0.555)
	White	1.51 (0.75–3.02, *p* = 0.247)	1.18 (0.57–2.45, *p* = 0.657)
	*Montubio or other*	1.05 (0.61–1.81, *p* = 0.865)	0.93 (0.51–1.67, *p* = 0.801)
Area of residence	Urban	Reference	Reference
	Rural	0.79 (0.64–0.98, *p* = 0.030)	1.06 (0.84–1.35, *p* = 0.615)
Region	Highlands	Reference	Reference
	Coast	1.11 (0.91–1.35, *p* = 0.305)	1.34 (1.07–1.69, *p* = 0.011)
	Amazon	0.76 (0.60–0.97, *p* = 0.026)	0.96 (0.76–1.23, *p* = 0.764)
	Insular region	1.89 (1.37–2.60, *p* < 0.001)	2.02 (1.42–2.88, *p* < 0.001)
Active commuting (days)	Per one additional day	0.98 (0.95–1.02, *p* = 0.423)	0.99 (0.95–1.03, *p* = 0.664)
Daily physical education (hour)	Per one additional hour	1.10 (0.91–1.33, *p* = 0.341)	1.06 (0.87–1.31, *p* = 0.557)
Weekly MVPA (days)	Per one additional day	0.96 (0.92–1.01, *p* = 0.088)	0.95 (0.90–0.99, *p* = 0.022)
Daily sedentary behavior (hours)	Per one additional hour	0.98 (0.91–1.06, *p* = 0.649)	0.99 (0.92–1.07, *p* = 0.842)
Daily fruit consumption status	0 servings	Reference	Reference
	1 serving	0.53 (0.18–1.53, *p* = 0.239)	0.66 (0.23–1.94, *p* = 0.453)
	2 servings	0.60 (0.21–1.67, *p* = 0.325)	0.80 (0.29–2.19, *p* = 0.659)
	3 or more servings	0.65 (0.24–1.77, *p* = 0.396)	0.71 (0.27–1.86, *p* = 0.489)
Daily vegetables consumption status	0 servings	Reference	Reference
	1 serving	0.30 (0.10–0.95, *p* = 0.041)	0.25 (0.08–0.77, *p* = 0.016)
	2 servings	0.30 (0.10–0.95, *p* = 0.040)	0.24 (0.08–0.76, *p* = 0.015)
	3 or more servings	0.36 (0.12–1.13, *p* = 0.081)	0.30 (0.10–0.93, *p* = 0.037)
Daily soft drinks consumption status	0 servings	Reference	Reference
	1 serving	1.55 (0.74–3.24, *p* = 0.246)	1.40 (0.67–2.90, *p* = 0.371)
	2 servings	1.59 (0.75–3.36, *p* = 0.224)	1.46 (0.70–3.05, *p* = 0.309)
	3 or more servings	1.21 (0.58–2.55, *p* = 0.611)	1.11 (0.53–2.31, *p* = 0.780)
Fast-food consumption (days)	Per one additional day	1.01 (0.94–1.08, *p* = 0.848)	1.06 (0.98–1.15, *p* = 0.128)
Processed-food consumption (days)	Per one additional day	1.00 (0.95–1.06, *p* = 0.955)	1.00 (0.94–1.06, *p* = 0.977)
Tooth brushing status	Nondaily	Reference	Reference
	Daily	1.12 (0.80–1.57, *p* = 0.501)	1.14 (0.80–1.62, *p* = 0.474)

*CI* confidence interval, *MVPA* moderate-to-vigorous physical activity, *OR* odds ratio.

## Discussion

Overall, a high prevalence of obesity was found in Ecuadorian children and adolescents, with more than one out of 10 participants having obesity. This high prevalence of obesity is in line with that reported in other international studies, both for South America and Ecuador.^1,2^ Supporting this notion, the last ENSANUT-2012 reported a lower prevalence of obesity (10.9%) than that observed in this study (12.7%), which indicates a growing upward trend. Although obesity in childhood and adolescence cannot be attributed to a single modifiable factor,^6,26^ this study further identified several factors related to obesity.

Concerning the factors related to obesity in Ecuadorian children and adolescents, the findings obtained highlight some inequalities in obesity according to sociodemographic factors. For instance, the results revealed that girls were less likely to have obesity than boys were. The same trend was also identified in the analyses stratified by age group. Although these findings were not consistent in LMICs, the prevalence of obesity is generally greater among boys than girls in most countries.^1^ Previous studies have shown that sex may be related to body weight and body composition patterns, with girls showing greater concerns for body image and a greater tendency to engage in weight loss behaviors to try to reach a normal weight,^27,28^ which could  (at least partially) explain our findings. In addition, sociocultural factors, such as social pressure and beauty ideals, may lead girls to be more conscious of their body image and exhibit behaviors related to weight loss.^29^ However, it is important to note that the prevalence of obesity varies according to other sociodemographic factors (i.e., age, race/ethnicity, or socioeconomic status).

Additionally, adolescents (aged 12 to 17 years) were less likely to have obesity than children (aged 5 to 11 years). These results were also observed in the analyses stratified by sex. A previous systematic review by Hajri et al.,^4^ which reported a slightly greater prevalence of obesity in children (10.7%) than in adolescents (10.5%) in Ecuador. However, other studies in other countries have reported a greater prevalence of obesity in adolescents than in children. For instance, one study in the United States reported that the prevalence of obesity among adolescents (aged 12 to 19 years; 20.6%) was greater than that among school-aged children (aged 6 to 11 years; 18.4%). Similarly, another study carried out in China revealed a greater prevalence of obesity in children (aged 7 to 12 years; 20.3%) than in adolescents (aged 13 to 17 years; 9.6%).^30^ During adolescence, there is an increased emphasis on matters related to one’s appearance, body weight, and various psychological aspects of development,^31^ which can explain these results. Another possible reason is that adolescents may have a better understanding of the consequences of unhealthy lifestyles, leading them to make more informed choices about their diet and physical activity compared to children.^32^

Furthermore, our results revealed an inverse relationship between the wealth family index and the prevalence of obesity. This result is in line with a previous study conducted by Abril et al.^33^ in children aged 6 to 9 years from Cuenca (Ecuador). In addition, a systematic review showed that European children whose parents were of greater socioeconomic status had a lower likelihood of having overweight or obesity.^34^ Similarly, Kim et al.^35^ reported in their longitudinal study in the United States that children from families with low incomes were more likely to have obesity than their counterpart children from families with high incomes were. Furthermore, another prospective cohort study by Wang et al.^36^ reported that poverty status was found to be the strongest predictor of obesity over time in youth from United States. Interestingly, a review by Vazquez and Cubbin^12^ outlined a clear pattern of an inverse relationship between socioeconomic status and childhood obesity in high-income countries.^12^ However, these same authors reported a disproportionate representation of research on the relationship between socioeconomic status and obesity in more affluent countries and, given the differential association between socioeconomic status and obesity-related outcomes in LMICs.^12^ In this sense, this study contributes to reducing the knowledge gap between studies conducted in high-income countries and those conducted in LMICs (i.e., Ecuador).

A higher education level of the breadwinner (i.e., middle/high school or higher) was also related to a lower probability of having obesity. This result is in line with the literature indicating that children of parents with higher education levels are less likely to have obesity.^37^ In this sense, for children with a low family income, children whose parents had less education were more likely to be in the trajectory of the obesity group than their counterparts were.^35^ Higher education levels are associated with healthier and more diverse diets, marked by increased consumption of fibers, fruits, vegetables, and fish products, whereas lower educational attainment tends to be associated with diets high in carbohydrates, sweets, and red meats, coupled with higher energy intake and larger portion sizes,^38^ which could be related to obesity among children and adolescents.^39^ Another possible reason is that education level may be a factor leading to greater leisure-time physical activity,^40^ which has been related to lower odds of having obesity.^13,39,41,42^ These findings are also in line with the results observed for the wealth family index, which is closely related to education level. In this sense, a study reported that the association between fathers’ and mothers’ education levels and obesity is moderated by household wealth.^43^ This could explain, at least in part, why higher levels of both factors were associated with a lower probability of obesity in Ecuadorian children and adolescents.

Another interesting finding was that participants from the coast and insular region (i.e., *Islas* *Galápagos*) were more likely to have obesity than their counterparts from the highlands. Despite the variability that exists between the different regions of the countries, this result is in line with the literature that points to differences depending on the region where children and adolescents live.^44,45^ One possible explanation is that the coast and insular region may have different dietary patterns than the highlands. Access to different types of food, availability of fresh produce, and cultural eating habits can vary between regions and influence the nutritional choices of individuals.^46^ Furthermore, sociocultural factors, including cultural norms and attitudes toward body image, physical activity, and diet, can vary across regions (i.e., the coast or insular region versus the highlands). These factors could impact lifestyle choices and contribute to disparities in obesity prevalence.^47^

Importantly, a greater number of days spent in physical activity was related to a lower probability of having obesity. This finding is in line with the scientific literature indicating the benefits of engaging in sufficient physical activity on obesity-related indicators.^13,39,41,42^ For instance, a systematic review with a meta-analysis by Poorolajal et al.^39^ with 1,636,049 children and adolescents reported lower odds of having obesity in those with sufficient physical activity. Similarly, García-Hermoso et al.^42^ reported that adolescents from the United States who met physical activity recommendations (especially in combination with meeting muscle‐strengthening activity recommendations) were less likely to have obesity. In addition, the low prevalence of meeting physical activity recommendations identified in this study in Ecuadorian children and adolescents is in line with the findings of previous studies,^48,49^ in Latin America, and in Ecuador.^50^ Given the low prevalence of physical activity in Ecuadorian children and adolescents, as well as its potential role in the prevention of childhood and adolescent obesity, initiatives to increase both the level of physical activity and equal opportunities for its practice in this age group are necessary.^20^

This study also revealed lower odds of having obesity in Ecuadorian children and adolescents who consume vegetables daily, which agrees with the findings of the scientific literature.^16,51–53^ For instance, a systematic review of cohort studies revealed that increasing vegetable intake reduces the risk of weight gain, overweight or obesity (in adults).^53^ More specifically, Ma et al.^52^ reported that greater vegetable consumption is related to a lower risk of cardiometabolic risk clusters (including obesity) in Chinese children and adolescents. Supporting this notion, the World Health Organization states that, when consumed as part of a healthy diet, vegetables (and fruits) may help prevent weight gain and reduce the risk of obesity among children and adolescents for various reasons.^54^ First, vegetables have a low caloric content and high fiber content, aiding in weight management by creating a sense of fullness and decreasing overall energy intake. Second, their abundance of essential nutrients can contribute to overall well-being and growth, potentially lowering the likelihood of complications associated with obesity. Third, when integrated into a nutritious diet that is low in fats, sugars, and salt, vegetables can assist in averting weight gain and diminishing the risk of obesity (among other noncommunicable diseases).

This study has several limitations. First, given the cross-sectional design of the study, we are not able to determine neither a cause‒effect relationship between the variables examined nor the direction of their association. Therefore, more longitudinal and experimental studies should be conducted to determine these relationships. Second, the questions for data collection in the ENSANUT-2018 were generally brief (intended to reduce the question burden on participants) and did not provide in-depth information to better understand the study findings. A more detailed measure would provide additional information for each item, as well as information on other aspects related to sociodemographic and lifestyle variables. Third, lifestyle data may lead to differential bias due to recall and information bias, social desirability bias, or underestimation/overestimation by parents/guardians or adolescents. Fourth, although we included relevant obesity-related factors, including sociodemographic factors (age, sex, race/ethnicity, wealth index, educational level, area of residence, region) and lifestyle variables (eating habits, physical activity, physical education, tooth brushing), it is still possible that other factors were involved. Conversely, this study has several strengths. The main strength of this study is the use of a nationwide and representative sample of children and adolescents from Ecuador, which provides external validity to the results, increasing the applicability and representativeness of the real-world scenarios. Furthermore, to our knowledge, this is the first study to examine sociodemographic and lifestyle variables related to obesity in a nationally representative sample of Ecuadorian children and adolescents (aged 5 to 17 years).

In conclusion, the high prevalence of obesity in Ecuadorian children and adolescents is a public health concern. Inequalities in sociodemographic factors (i.e., age, sex, wealth, educational level of the main breadwinner, and region), as well as in lifestyle factors (i.e., physical activity, vegetable consumption), seem to play a crucial role in the prevalence of obesity. These results highlight the need to consider sociodemographic and lifestyle factors in the prevention and management of obesity in Ecuador. Policies need to be implemented that address inequalities in obesity prevention and control, especially for younger children, boys, those from poorer families, those with a breadwinner with low educational level, and  for those living on the coast or in the insular region. These measures may involve advocating for the affordability and accessibility of vegetables and establishing secure and convenient areas for engaging in physical activities.

## Supplementary information

Supplementary material

Figure S1

## Data Availability

The datasets analyzed during the current study are available from the corresponding author upon reasonable request.
