# Supplementary material for: Prevalence of obesity and associated sociodemographic and lifestyle factors in Ecuadorian children and adolescents
Source: Pediatr Res. 2024 Jun 24;97(1):422–9. doi: 10.1038/s41390-024-03342-w (PMC11798822; doi:10.1038/s41390-024-03342-w)
Supplement: Supplementary file 1 — Supplementary material [file 41390_2024_3342_MOESM1_ESM.pdf]

Supplementary material

Table S1. Survey-weighted generalized linear models to evaluate the probability of having obesity in the Ecuadorian boys 5- to 17-year-old of the *Encuesta Nacional de Salud y Nutrición*, 2018.

		Dependent variable (obesity)	
		Univariate analysis	*Multivariate analysis*
Predictors	*Levels*	*OR (95% CI, p value)*	*OR (95% CI, p value)*
Age group	Children (5 to 11 years)	Reference	Reference
	Adolescents (12 to 17 years)	**0.40 (0.31-0.53, *p*<0.001)**	**0.49 (0.37-0.67, *p*<0.001)**
Educational level of the main breadwinner	Primary education or lower	Reference	Reference
	Middle/high school or higher	**0.25 (0.13-0.48, *p*<0.001)**	**0.38 (0.19-0.78, *p*=0.008)**
Wealth family index status	Very rich	Reference	Reference
	Rich	0.99 (0.66-1.50, *p*=0.969)	0.98 (0.63-1.51, *p*=0.921)
	Medium	**1.92 (1.28-2.89, *p*=0.002)**	**2.02 (1.29-3.16, *p*=0.002)**
	Poor	**1.86 (1.23-2.81, *p*=0.003)**	**1.98 (1.22-3.23, *p*=0.006)**
	Very poor	**1.91 (1.26-2.91, *p*=0.003)**	**2.23 (1.33-3.75, *p*=0.002)**
Race/ethnicity	White	Reference	Reference
	Afro-Ecuadorian	0.81 (0.31-2.10, *p*=0.659)	0.78 (0.30-2.02, *p*=0.602)
	Mestizo	0.86 (0.39-1.93, *p*=0.723)	0.79 (0.35-1.75, *p*=0.558)
	Indigenous	0.64 (0.27-1.52, *p*=0.311)	0.78 (0.32-1.93, *p*=0.595)
	Montubio or other	0.68 (0.26-1.79, *p*=0.437)	0.76 (0.29-2.00, *p*=0.575)
Area of residence	Urban	Reference	Reference
	Rural	**0.73 (0.55-0.97, *p*=0.032)**	1.03 (0.74-1.43, *p*=0.875)
Region	Highlands	Reference	Reference
	Coast	1.02 (0.79-1.33, *p*=0.874)	1.24 (0.92-1.68, *p*=0.155)
	Amazon	**0.66 (0.49-0.90, *p*=0.009)**	0.84 (0.61-1.15, *p*=0.278)
	Insular region	**1.74 (1.15-2.62, *p*=0.008)**	**1.98 (1.23-3.19, *p*=0.005)**
Active commuting (days)	Per one additional day	1.00 (0.95-1.05, *p*=0.897)	1.02 (0.97-1.07, *p*=0.547)
Daily physical education (hour)	Per one additional hour	1.16 (0.90-1.50, *p*=0.239)	1.12 (0.85-1.46, *p*=0.418)

Predictors	Levels	*Dependent variable (obesity)*<br>*Univariate analysis*<br>*OR (95% CI, p value)*	*Multivariate analysis*<br>*OR (95% CI, p value)*
Weekly MVPA (days)	Per one additional day	0.95 (0.90-1.00, *p*=0.072)	0.96 (0.90-1.01, *p*=0.124)
Daily sedentary behavior (hours)	Per one additional hour	0.97 (0.88-1.06, *p*=0.472)	0.96 (0.86-1.06, *p*=0.400)
Daily fruit consumption status	0 servings	Reference	Reference
	1 serving	0.30 (0.07-1.22, *p*=0.092)	**0.27 (0.07-0.99, *p*=0.049)**
	2 servings	0.37 (0.10-1.47, *p*=0.159)	0.37 (0.11-1.24, *p*=0.107)
	3 or more servings	0.46 (0.12-1.75, *p*=0.255)	0.35 (0.11-1.13, *p*=0.079)
Daily vegetables consumption status	0 servings	Reference	Reference
	1 serving	0.83 (0.10-7.08, *p*=0.865)	0.85 (0.10-6.96, *p*=0.880)
	2 servings	0.77 (0.09-6.49, *p*=0.810)	0.67 (0.08-5.32, *p*=0.704)
	3 or more servings	1.16 (0.14-9.72, *p*=0.888)	1.05 (0.13-8.28, *p*=0.965)
Daily soft drinks consumption status	0 servings	Reference	Reference
	1 serving	2.09 (0.86-5.10, *p*=0.104)	1.82 (0.68-4.87, *p*=0.235)
	2 servings	1.87 (0.77-4.59, *p*=0.169)	1.56 (0.58-4.19, *p*=0.377)
	3 or more servings	1.56 (0.65-3.76, *p*=0.323)	1.41 (0.53-3.79, *p*=0.490)
Fast-food consumption (days)	Per one additional day	1.02 (0.93-1.13, *p*=0.676)	1.06 (0.96-1.17, *p*=0.274)
Processed-food consumption (days)	Per one additional day	1.03 (0.96-1.10, *p*=0.393)	1.02 (0.96-1.09, *p*=0.501)
Tooth brushing status	Nondaily	Reference	Reference
	Daily	0.96 (0.62-1.47, *p*=0.835)	0.93 (0.59-1.46, *p*=0.741)

CI, confidence interval; MVPA, moderate-to-vigorous physical activity; OR, odds ratio.

Table S2. Survey-weighted generalized linear models to evaluate the probability of having obesity in the Ecuadorian girls 5- to 17-year-old of the *Encuesta Nacional de Salud y Nutrición*, 2018.

		Dependent variable (obesity)	
		Univariate analysis	*Multivariate analysis*
Predictors	*Levels*	*OR (95% CI, p value)*	*OR (95% CI, p value)*
Age group	Children (5 to 11 years)	Reference	Reference
	Adolescents (12 to 17 years)	**0.55 (0.40-0.75, *p*<0.001)**	**0.51 (0.36-0.74, *p*<0.001)**
Educational level of the main breadwinner	Primary education or lower	Reference	Reference
	Middle/high school or higher	0.68 (0.41-1.12, *p*=0.128)	0.97 (0.54-1.75, *p*=0.925)
Wealth family index status	Very rich	Reference	Reference
	Rich	1.24 (0.70-2.19, *p*=0.461)	1.28 (0.71-2.30, *p*=0.407)
	Medium	0.96 (0.56-1.64, *p*=0.875)	1.07 (0.61-1.87, *p*=0.809)
	Poor	1.13 (0.65-1.97, *p*=0.665)	1.32 (0.73-2.40, *p*=0.364)
	Very poor	1.47 (0.84-2.57, *p*=0.175)	1.78 (0.94-3.35, *p*=0.075)
Race/ethnicity	White	Reference	Reference
	Afro-Ecuadorian	0.66 (0.23-1.92, *p*=0.451)	0.73 (0.24-2.19, *p*=0.576)
	Mestizo	1.28 (0.54-3.02, *p*=0.580)	1.40 (0.58-3.35, *p*=0.455)
	Indigenous	0.82 (0.29-2.34, *p*=0.713)	1.08 (0.35-3.28, *p*=0.897)
	Montubio or other	0.81 (0.26-2.58, *p*=0.728)	0.90 (0.26-3.09, *p*=0.863)
Area of residence	Urban	Reference	Reference
	Rural	0.87 (0.60-1.26, *p*=0.470)	1.14 (0.74-1.75, *p*=0.548)
Region	Highlands	Reference	Reference
	Coast	1.27 (0.91-1.76, *p*=0.166)	**1.56 (1.10-2.21, *p*=0.013)**
	Amazon	0.94 (0.64-1.37, *p*=0.739)	1.18 (0.80-1.73, *p*=0.406)
	Insular region	**2.15 (1.33-3.48, *p*=0.002)**	**2.20 (1.28-3.78, *p*=0.004)**
Active commuting (days)	Per one additional day	0.96 (0.90-1.02, *p*=0.189)	0.96 (0.90-1.03, *p*=0.286)
Daily physical education (hour)	Per one additional hour	0.98 (0.73-1.31, *p*=0.894)	0.99 (0.72-1.35, *p*=0.926)
Weekly MVPA (days)	Per one additional day	0.95 (0.88-1.02, *p*=0.190)	0.95 (0.88-1.02, *p*=0.179)
Daily sedentary behavior (hours)	Per one additional hour	1.01 (0.90-1.14, *p*=0.826)	1.05 (0.93-1.18, *p*=0.459)

		Dependent variable (obesity)	
		Univariate analysis	*Multivariate analysis*
Predictors	*Levels*	*OR (95% CI, p value)*	*OR (95% CI, p value)*
Daily fruit consumption status	0 servings	Reference	Reference
	1 serving	1.51 (0.31-7.45, *p*=0.612)	4.23 (0.94-19.10, *p*=0.061)
	2 servings	1.52 (0.31-7.43, *p*=0.606)	3.87 (0.88-16.97, *p*=0.073)
	3 or more servings	1.39 (0.30-6.53, *p*=0.676)	3.16 (0.78-12.72, *p*=0.106)
Daily vegetables consumption status	0 servings	Reference	Reference
	1 serving	**0.23 (0.06-0.85, *p*=0.027)**	**0.13 (0.03-0.50, *p*=0.003)**
	2 servings	**0.26 (0.07-0.96, *p*=0.043)**	**0.16 (0.04-0.61, *p*=0.007)**
	3 or more servings	**0.23 (0.06-0.82, *p*=0.023)**	**0.16 (0.04-0.57, *p*=0.005)**
Daily soft drinks consumption status	0 servings	Reference	Reference
	1 serving	1.08 (0.37-3.19, *p*=0.883)	1.16 (0.40-3.39, *p*=0.785)
	2 servings	1.36 (0.45-4.13, *p*=0.583)	1.53 (0.52-4.53, *p*=0.438)
	3 or more servings	0.91 (0.31-2.69, *p*=0.864)	0.99 (0.33-2.93, *p*=0.986)
Fast-food consumption (days)	Per one additional day	0.99 (0.89-1.10, *p*=0.816)	1.06 (0.93-1.20, *p*=0.377)
Processed-food consumption (days)	Per one additional day	0.97 (0.88-1.08, *p*=0.586)	0.97 (0.88-1.08, *p*=0.616)
Tooth brushing status	Nondaily	Reference	Reference
	Daily	1.73 (0.99-3.04, *p*=0.056)	1.81 (0.96-3.42, *p*=0.066)

CI, confidence interval; MVPA, moderate-to-vigorous physical activity; OR, odds ratio.Ê

Table S3. Survey-weighted generalized linear models to evaluate the probability of having obesity in the Ecuadorian 5- to 11-year-old population of the *Encuesta Nacional de Salud y Nutrición*, 2018.

		Dependent variable (obesity)	
		Univariate analysis	*Multivariate analysis*
Predictors	*Levels*	*OR (95% CI, p value)*	*OR (95% CI, p value)*
Sex	Boys	Reference	Reference
	Girls	**0.63 (0.50-0.78, *p*<0.001)**	**0.58 (0.46-0.72, *p*<0.001)**
Educational level of the main breadwinner	Primary education or lower	Reference	Reference
	Middle/high school or higher	1.00 (1.00-1.00, *p*=1.000)	1.00 (1.00-1.00, *p*=1.000)
Wealth family index status	Very rich	Reference	Reference
	Rich	1.34 (0.91-1.96, *p*=0.141)	1.35 (0.90-2.02, *p*=0.152)
	Medium	**1.98 (1.36-2.89, *p*<0.001)**	**1.98 (1.31-2.99, *p*=0.001)**
	Poor	**1.80 (1.24-2.63, *p*=0.002)**	**1.74 (1.12-2.70, *p*=0.014)**
	Very poor	**2.43 (1.63-3.62, *p*<0.001)**	**2.35 (1.45-3.80, *p*=0.001)**
Race/ethnicity	White	Reference	Reference
	Afro-Ecuadorian	0.60 (0.24-1.46, *p*=0.259)	0.63 (0.25-1.54, *p*=0.307)
	Mestizo	0.82 (0.38-1.76, *p*=0.605)	0.78 (0.36-1.71, *p*=0.534)
	Indigenous	0.54 (0.23-1.30, *p*=0.171)	0.66 (0.26-1.65, *p*=0.369)
	Montubio or other	0.61 (0.24-1.57, *p*=0.306)	0.71 (0.27-1.86, *p*=0.487)
Area of residence	Urban	Reference	Reference
	Rural	0.78 (0.61-0.99, *p*=0.042)	1.01 (0.76-1.34, *p*=0.938)
Region	Highlands	Reference	Reference
	Coast	0.93 (0.74-1.17, *p*=0.537)	1.16 (0.89-1.53, *p*=0.273)
	Amazon	0.77 (0.58-1.00, *p*=0.053)	1.04 (0.78-1.38, *p*=0.790)
	Insular region	**2.18 (1.46-3.25, *p*<0.001)**	**2.49 (1.58-3.93, *p*<0.001)**
Active commuting (days)	Per one additional day	**0.95 (0.91-0.99, *p*=0.020)**	0.97 (0.93-1.02, *p*=0.263)
Daily physical education (hour)	Per one additional hour	1.01 (0.79-1.28, *p*=0.961)	0.95 (0.73-1.23, *p*=0.711)
Weekly MVPA (days)	Per one additional day	**0.93 (0.89-0.98, *p*=0.005)**	**0.94 (0.89-0.98, *p*=0.008)**
Daily sedentary behavior (hours)	Per one additional hour	1.04 (0.95-1.14, *p*=0.407)	1.01 (0.91-1.11, *p*=0.877)

Predictors	*Levels*	*Dependent variable (obesity)*<br>*Univariate analysis*<br>*OR (95% CI, p value)*	 *Multivariate analysis*<br>*OR (95% CI, p value)*
Daily fruit consumption status	0 servings	Reference	Reference
	1 serving	0.48 (0.12-1.89, *p*=0.297)	0.75 (0.21-2.71, *p*=0.666)
	2 servings	0.50 (0.13-1.90, *p*=0.306)	0.80 (0.24-2.68, *p*=0.716)
	3 or more servings	0.61 (0.16-2.27, *p*=0.459)	0.83 (0.26-2.61, *p*=0.751)
Daily vegetables consumption status	0 servings	Reference	Reference
	1 serving	**0.24 (0.07-0.77, *p*=0.017)**	**0.16 (0.05-0.54, *p*=0.003)**
	2 servings	**0.24 (0.07-0.76, *p*=0.016)**	**0.16 (0.05-0.51, *p*=0.002)**
	3 or more servings	**0.30 (0.09-0.96, *p*=0.043)**	**0.17 (0.05-0.57, *p*=0.004)**
Daily soft drinks consumption status	0 servings	Reference	Reference
	1 serving	1.41 (0.52-3.84, *p*=0.496)	1.76 (0.63-4.95, *p*=0.284)
	2 servings	1.99 (0.73-5.37, *p*=0.176)	2.39 (0.85-6.69, *p*=0.098)
	3 or more servings	1.44 (0.53-3.95, *p*=0.476)	1.63 (0.58-4.61, *p*=0.356)
Fast-food consumption (days)	Per one additional day	1.04 (0.96-1.12, *p*=0.394)	1.05 (0.95-1.15, *p*=0.351)
Processed-food consumption (days)	Per one additional day	1.04 (0.97-1.11, *p*=0.238)	1.04 (0.98-1.12, *p*=0.209)
Tooth brushing status	Nondaily	Reference	Reference
	Daily	1.18 (0.80-1.74, *p*=0.396)	1.14 (0.76-1.69, *p*=0.524)

CI, confidence interval; MVPA, moderate-to-vigorous physical activity; OR, odds ratio. † Given that 100% of the participants included had “Primary Education or lower”, this variable was not included in the model.

Table S4. Survey-weighted generalized linear models to evaluate the probability of having obesity in the Ecuadorian 12- to 17-year-old population of the *Encuesta Nacional de Salud y Nutrición*, 2018.

		Dependent variable (obesity)	
		Univariate analysis	*Multivariate analysis*
Predictors	*Levels*	*OR (95% CI, p value)*	*OR (95% CI, p value)*
Sex	Boys	Reference	Reference
	Girls	0.86 (0.59-1.25, *p*=0.417)	0.85 (0.57-1.25, *p*=0.403)
Educational level of the main breadwinner	Primary education or lower	Reference	Reference
	Middle/high school or higher	**0.63 (0.41-0.98, *p*=0.040)**	0.67 (0.42-1.05, *p*=0.082)
Wealth family index status	Very rich	Reference	Reference
	Rich	0.78 (0.38-1.58, *p*=0.489)	0.79 (0.39-1.58, *p*=0.504)
	Medium	0.84 (0.42-1.70, *p*=0.635)	0.92 (0.47-1.83, *p*=0.821)
	Poor	1.19 (0.61-2.32, *p*=0.613)	1.32 (0.68-2.55, *p*=0.414)
	Very poor	0.99 (0.52-1.89, *p*=0.968)	1.36 (0.66-2.83, *p*=0.404)
Race/ethnicity	White	Reference	Reference
	Afro-Ecuadorian	1.01 (0.32-3.15, *p*=0.987)	1.22 (0.39-3.85, *p*=0.736)
	Mestizo	1.51 (0.61-3.74, *p*=0.370)	1.64 (0.64-4.18, *p*=0.299)
	Indigenous	1.21 (0.44-3.35, *p*=0.716)	1.66 (0.55-5.05, *p*=0.371)
	Montubio or other	1.00 (0.28-3.57, *p*=0.997)	0.93 (0.24-3.55, *p*=0.917)
Area of residence	Urban	Reference	Reference
	Rural	0.90 (0.63-1.30, *p*=0.583)	1.15 (0.75-1.76, *p*=0.526)
Region	Highlands	Reference	Reference
	Coast	**1.47 (1.02-2.10, *p*=0.036)**	**1.78 (1.19-2.67, *p*=0.005)**
	Amazon	0.75 (0.48-1.15, *p*=0.186)	0.80 (0.52-1.25, *p*=0.333)
	Insular region	1.50 (0.82-2.75, *p*=0.189)	1.31 (0.69-2.50, *p*=0.405)
Active commuting (days)	Per one additional day	1.04 (0.97-1.12, *p*=0.257)	1.03 (0.96-1.11, *p*=0.376)
Daily physical education (hour)	Per one additional hour	1.35 (0.95-1.92, *p*=0.093)	1.22 (0.85-1.74, *p*=0.279)
Weekly MVPA (days)	Per one additional day	1.00 (0.91-1.10, *p*=0.987)	0.99 (0.90-1.09, *p*=0.847)
Daily sedentary behavior (hours)	Per one additional hour	0.99 (0.87-1.12, *p*=0.839)	1.01 (0.89-1.15, *p*=0.885)

		Dependent variable (obesity)	
		Univariate analysis	*Multivariate analysis*
Predictors	*Levels*	*OR (95% CI, p value)*	*OR (95% CI, p value)*
Daily fruit consumption status	0 servings	Reference	Reference
	1 serving	0.52 (0.09-3.05, *p*=0.468)	0.43 (0.07-2.89, *p*=0.388)
	2 servings	0.75 (0.13-4.25, *p*=0.745)	0.68 (0.12-3.86, *p*=0.662)
	3 or more servings	0.64 (0.12-3.47, *p*=0.601)	0.43 (0.08-2.37, *p*=0.335)
Daily vegetables consumption status	0 to 1 serving [†]	Reference	Reference
	2 servings	1.08 (0.59-1.99, *p*=0.803)	1.00 (0.47-2.12, *p*=0.995)
	3 or more servings	1.16 (0.71-1.90, *p*=0.560)	1.48 (0.68-3.22, *p*=0.325)
Daily soft drinks consumption status	0 servings	Reference	Reference
	1 serving	1.62 (0.57-4.56, *p*=0.363)	1.39 (0.49-3.92, *p*=0.534)
	2 servings	0.87 (0.29-2.62, *p*=0.798)	0.76 (0.25-2.28, *p*=0.621)
	3 or more servings	0.93 (0.33-2.57, *p*=0.882)	0.76 (0.27-2.15, *p*=0.608)
Fast-food consumption (days)	Per one additional day	1.04 (0.91-1.20, *p*=0.554)	1.11 (0.97-1.27, *p*=0.143)
Processed-food consumption (days)	Per one additional day	**0.90 (0.81-1.00, *p*=0.047)**	0.91 (0.82-1.02, *p*=0.091)
Tooth brushing status	Nondaily	Reference	Reference
	Daily	1.05 (0.49-2.23, *p*=0.908)	1.12 (0.52-2.43, *p*=0.769)

CI, confidence interval; MVPA, moderate-to-vigorous physical activity; OR, odds ratio. [†] Given that the "0 servings" category presented a very low number of cases, this category was merged with the "1 serving" category.Ȅ
