# Supplementary figures and images for: Prevalence of obesity and associated sociodemographic and lifestyle factors in Ecuadorian children and adolescents
Source: Pediatr Res. 2024 Jun 24;97(1):422–9. doi: 10.1038/s41390-024-03342-w (PMC11798822; doi:10.1038/s41390-024-03342-w)

**Figure S1.** Flowchart with the selection of study participants.

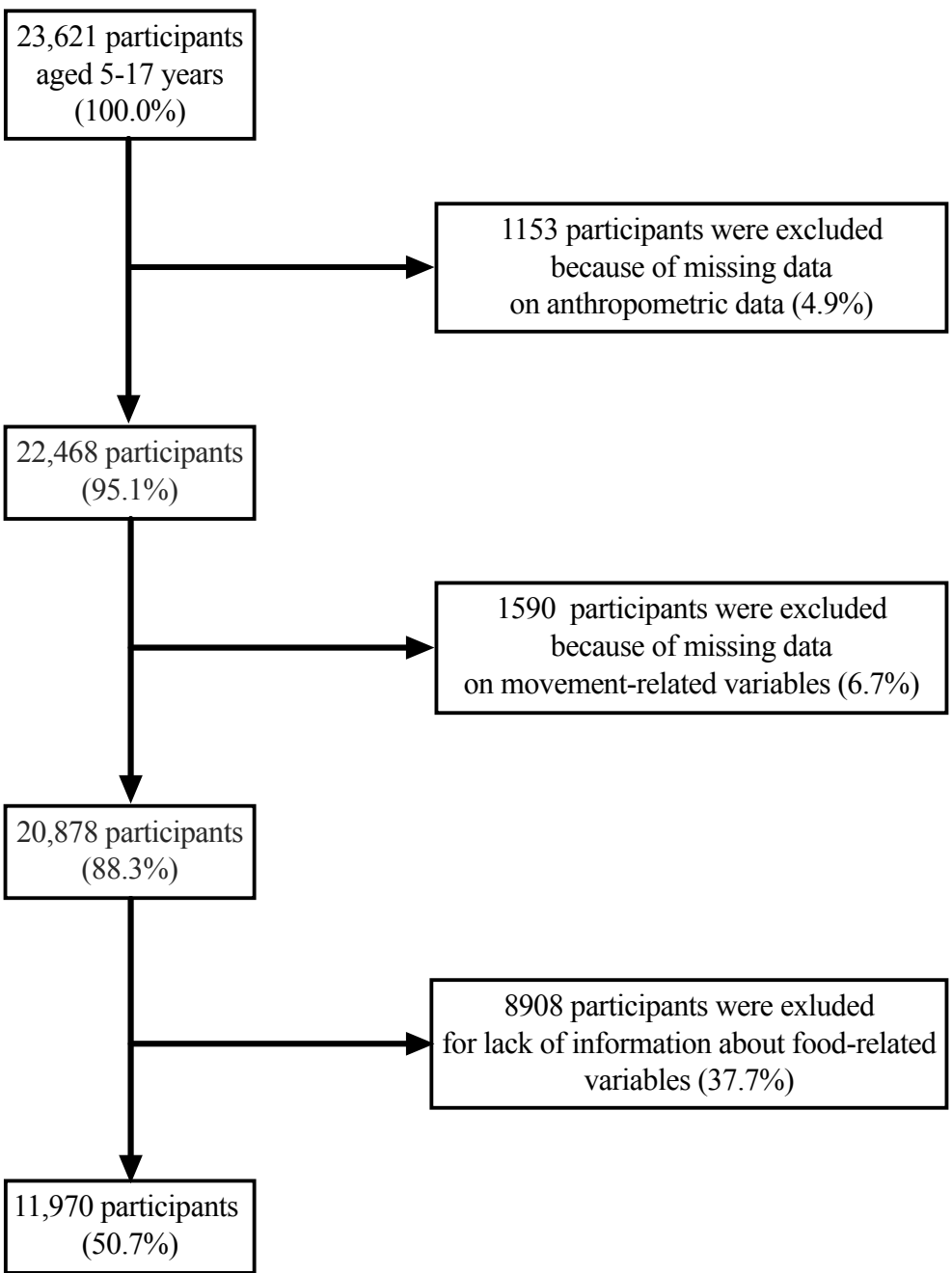

Supplement: Supplementary file 2 — Figure S1 [file 41390_2024_3342_MOESM2_ESM.pdf]
